# How Do R&D and Renewable Energy Consumption Lead to Carbon Neutrality? Evidence from G-7 Economies

**DOI:** 10.3390/ijerph20054604

**Published:** 2023-03-05

**Authors:** Qi Xu, Salim Khan

**Affiliations:** Business School, Zhengzhou University, Zhengzhou 450001, China

**Keywords:** research and development, renewable energy, CO_2_, CS-ARDL, G-7

## Abstract

The discussion about whether research and development and advanced energy structure can efficiently control pollution has gained the consideration of researchers across the globe. However, there is a lack of enough empirical and theoretical evidence to support this phenomenon. To offer support of empirical evidence along with theoretical mechanism, we examine the net Impact of research and development (R&D) and renewable energy consumption (RENG) on CO2E utilizing panel data from G-7 economies for 1990–2020. Moreover, this study investigates the controlling role of economic growth and nonrenewable energy consumption (NRENG) in the R&D-CO2E models. The results obtained from the CS-ARDL panel approach verified a long-run and short-run relationship between R&D, RENG, economic growth, NRENG, and CO2E. Short- and long-run empirical results suggest that R&D and RENG improve environmental stability by decreasing CO2E, while economic growth and NRENG increase CO2E. Particularly, long-run R&D and RENG reduce CO2E with the effect of −0.091 and −0.101, respectively, while in the short run, they reduce CO2E with the effect of −0.084 and −0.094, respectively. Likewise, the 0.650% (long run) and 0.700% (short-run) increase in CO2E is due to economic growth, while the 0.138% (long run) and 0.136% (short run) upsurge in CO2E is due to an increase in NRENG. The findings obtained from the CS-ARDL model were also verified by the AMG model, while D-H non-causality approach was applied to check the pair-wise relationship among variables. The D-H causal relationship revealed that policies to focus on R&D, economic growth, and NRENG explain variation in CO2E but not vice versa. Furthermore, policies considering RENG and human capital can also affect CO2E and vice versa, meaning there is a round effect between the variables. All this indication may guide the concerned authorities to devise comprehensive policies that are helpful to environmental stability and in line with CO2E reduction.

## 1. Introduction

Climate change due to carbon emission (CO2E) has become not only a scientific issue but also an international political and economic matter of concern. Since the beginning of the 21st century, the economy and society have entered a period of high development, and various social and ecological problems have been increasingly exposed. The Global Carbon Project predicts that global carbon dioxide emissions will reach 40.6 billion tons this year, almost equal to the total carbon emissions in 2019 and 5% higher than when the Paris Agreement was signed in 2015 [1,2,3]. Among them, carbon dioxide emissions (CO2E) related to fossil fuels will reach 36.6 billion tons, the highest on record [4]. Specifically, CO2E from the oil sector is likely to increase by more than 2% compared to last year, coal sector emissions will return to their historical peak in 2014, and natural gas sector emissions are expected to remain unchanged but at the same level as in 2021 [5]. It is worth noting that since 2000, methane emissions have augmented by 8.99% globally, while about fifty million tons per year and humans cause 60% of methane emissions, as reported by UN Environmental Program [6].

According to the latest United Nations Environment Program (UNEP), the global construction industry has vigorously increased investment in energy efficiency and reduced its energy intensity. Energy consumption and CO2E caused by buildings and construction still exceeded the level before the new crown pneumonia epidemic outbreak, hitting a record high [7,8,9]. Data show that the building and construction industry will account for more than 34% of global energy demand; among the carbon dioxide emissions related to energy consumption and process, its proportion will reach about 37% [10]. Energy-related operational emissions from the buildings and construction sector reached 10 GtCO2E last year, 5% above 2020 levels and 2% above the pre-COVID-19 peak in 2019 [11].

**Figure 1:**
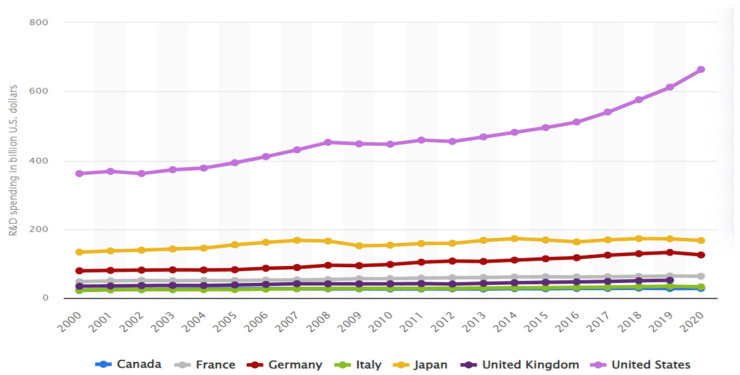
R&D trend in G7 economies (source: Statista 2020).

The synchronous growth in research and development (RD) increases economic growth, reduces CO2E, and increases the efficiency of clean energy production in various economic sectors [12]. In contrast, clean energy production, oil, natural gas, and other green energy use will reduce carbon emissions [13]. For example, from 1965 to the middle of the 19th century, the industrialization process of developed countries promoted rapid economic growth, and the total carbon emissions during this period also showed a fast upward trend [14]. During an economic recession, energy consumption and CO2E decline in stages have brought zero growth or decline in total carbon emissions [15,16]. However, in the economic recovery after the crisis, carbon emissions will show strong growth. International Energy Agency IEA [17] reports that the carbon emissions of the G-7 seven major economies in 2021 will increase to a certain extent compared with 2020. the development of CO2E fluctuates with economic growth and the energy structure adjustment; the relationship between CO2E growth and economic growth tends to weaken. (See visualize the trend of R&D in Figure 1 for G7 countries).

The current study contributes to the existing literature by analyzing the Impact of R&D, RENG, NRENG, and economic growth on CO2E. The G7 countries adopted a policy related to research and development that aims to enhance technological innovation and green growth and promote environmental sustainability at the regional level [18,19]. In addition, the policy adaptation aims to scientific knowledge and local research ability, generate a block for highly skilled people, and strengthen the process of advancing environmental science and research-oriented innovations and industries. The block of G7 economies also establishes a steady regional-level review of technological and research-based scientific systems (such as, technical R&D-based changes, R&D-based infrastructure, and industrial development) [20,21]. To cope with the uncertainty brought by the severe climate fluctuations and increasing CO2E, the block of G7 countries is trying to dynamically adjust the growth layout by strengthening the support of related research and innovation. To solve the problem facing the field of clean energy, thereby reducing climate change and energy influence on human well-being.

In protecting environmental sustainability, G7 economies inspected CO2E in its R&D, scientific adaptations, and publication-based publications policy to consider ecological stability [21]. The block of 7 countries has implemented various instrumental policies such as carbon tax policies, mitigative pollution policies, clean energy policies, and clean energy subsidies policies [22]. The adaptation of these Plans, the economic system of green and low-carbon cycles can be formed. The industrial structure, energy structure, and transportation structure could be made noticeable progress, and the efficiency of energy resource utilization and carbon emissions of the whole society would be continued to improve. The policymakers, therefore, must implement a plan that can keep net-zero CO2E by the end of 2030 [23,24,25,26].

Against the backdrop, the current study inspects how R&D, renewable and nonrenewable energy consumption, and economic growth influence environments in the form of CO2E. The analysis of the current study contributes to the existing literature in the following ways. First, this study disaggregates the primary explanatory variable and utilizes many factors related to research, development, and innovations. That is, to investigate the Impact of R&D (research per million) or the number of people involved in research and developmental projects and renewable energy consumption on CO2E. The selection procedures of the main explanatory variables are based on the existing literature and G7 criteria of innovation and technology. Notably, the researcher per million engaged in developing and devising ideas and policies, developmental techniques, technological-based instrumentation, and operational software and models. Furthermore, R&D covers basic research, applied research, and experimental development [27,28].

**Figure 2:**
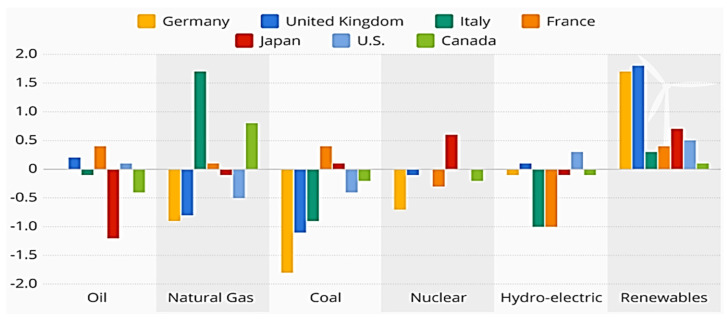
Sources of renewable energy in G7 economies (source: Statista 2019).

In addition to the above discussion, renewable energy consumption and production also involve the utilization of R&D-based projects, i.e., clean-energy technologies, which is a feasible substitute for nonrenewable energy consumption (see Figure 2). The different sources of renewable energy consumption in G7 countries). There are various justifications for inspecting the impact of renewable energy consumption of CO2E in the case of G7 economies. Adopting R&D and technology-based energy structures is essential in increasing economic growth and reducing CO2E [29,30,31]. However, renewable energy structure needs investment in research and innovation projects because of its dominant role in global green development and environmental stability [32]. Since the work of Wang, Zhang [33], and other empirical-based studies revealed a crucial role of renewable energy in achieving sustainable development goals (SDGs). Substituting renewable energy consumption with non-renewable energy would greatly and positively impact carbon neutrality. The existing literature indicates the permanent position of renewable energy consumption in mitigating environmental pollution and sustainable development.

Third, all G7 economies are well-developed and hold the highest economic growth, subsequently, higher energy consumption; therefore, they are required to enhance the quality of energy supply structure and recognize the pathways to technological-based energy for production and consumption [34]. Furthermore, sources of renewable energy supply are not used or are still in the development process in some parts of the G7 economies. Recognizing and utilizing those sources would help show the pathway of sustainable development and contribute to adopting a pollution mitigation policy [35]. Finally, the current research deployed the Cross-sectional Dependence Autoregressive Distributive Lag (CS-ARDL) advanced econometric model to address the long-run and short-run Impact of R&D, RECO, and other control variables on CO2E. The literature review part covers the following research paper in tabulated form.

To briefly summarize, the novelty of this paper lies in its examination of the interplay between research and development (R&D), renewable energy consumption (RENG), economic growth, nonrenewable energy consumption (NRENG), and CO_2_ emissions (CO2E) using panel data from the G-7 economies from 1990 to 2020. While previous studies have separately investigated the impact of R&D and renewable energy on CO2E, this paper combines them to explore their collective implications for environmental stability. Additionally, the study examines the role of economic growth and nonrenewable energy consumption in the R&D-CO2E models, which has not been widely explored in previous research. The paper utilizes the CS-ARDL panel approach and the D-H non-causality approach to examine the long-run and short-run relationships between these variables and their impact on environmental stability, providing a comprehensive analysis of the issue. The study’s findings offer valuable insights into the design of comprehensive policies that consider multiple factors and are effective in reducing CO2E and promoting environmental stability.

The remaining research sections are organized in the following way: Detailed literature and its Tabulated form of related studies (literature review) in Section 2. Material and method discuss in Section 3, Results and discussions in Section 4, and the Conclusion with its policy recommendation is covered in Section 5.

## 2. Literature Review

The study of the relationship between various environmental and economic variables is relevant to regional future economic development and social development. A large body of literature around the world uses different methods to study environment-economic variables, such as R&D, renewable energy, economic growth, nonrenewable energy consumption, and CO2E.

Adedoyin, Alola [36] examined the impact of R&D expenditure on environmental sustainability and investigated the long-run and causal relationship between RENG, economic growth, and ecological footprint. The study revealed a significant negative relationship between R&D expenditure and ecological footprint in the long run. This implies that spending on R&D significantly impacts the environmental sustainability of the panel countries. Additionally, the study affirms that NRENG and economic growth increase CO2E flaring, while RENG declines ecological footprint.

Similarly, the empirical findings of Alam, Apergis [37] indicated that there is a significant long-term equilibrium connection between R&D with both clean energy consumption and CO_2_ emissions. Furthermore, the long-run elasticities reveal that R&D and stock market growth favorably influence clean-energy consumption, whereas they have an unfavorable impact on CO2E.

This part of the study cannot offer a detailed literature review on R&D-CO2E, (non)renewable energy consumption-CO2E nexus, and economic growth-CO2E nexus. Therefore, the literature review part covers the following research paper in tabulated form, as shown in Table 1.

## 3. Materials and Methods

In the present study, firstly, we inspected the cross-sectional dependence (CSDP) rising from residuals’ mutuality, interdependence, unforeseen economic and environmental instability, and other shocks in numerous macro or micro-level socioeconomic factors. If the problem of CSDP is not tackled correctly, the study results would be considered partial [54]. Therefore, the famous test of Pesaran [55] CSDP approach is employed. Inspecting the problem of CSDP of all variables, the next step is to check the stationarity level of all the series.

**Figure 3:**
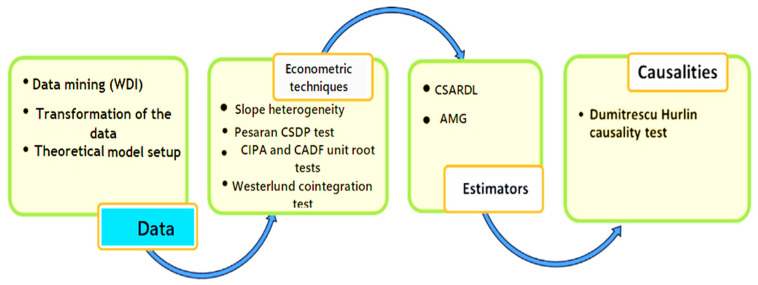
Methodological process.

After that, our study detects the existence of slope heterogeneity with the help of Pesaran and Yamagata [56]. We test the null hypothesis (H0) (slope homogeneity exists) against the H1. It is also worth considering that if the heterogeneity in slope is overlooked in the panel analysis, the empirical findings would be observed as unreliable and biased at later stages. The 3rd stage is determining whether the variables have a long-term connection; however, integrating CSDP is vital for this test. According to Westerlund and Edgerton [57], the panel cointegration tests report the CSDP, the problem of slope homogeneity, and serial correlation in error terms. We use the CS-ARDL to handle CSDP and slope heterogeneity after proving cointegration between study variables. See Figure 3 for the complete flowchart of the methodological process.

The variables of interest are; Carbon emissions (CO2E = dependent variable), research and development (R&D = main explanatory or independent variable), renewable energy consumption (RERC = main control variable), economic growth (GDP = main control variable), and nonrenewable energy consumption (NRERC control variable). s used in the above equation were converted to log form, including all control variables. The detail is mentioned in Table 2. All the variables of the study are specified as follows:

(1) $$\mathrm{CO}2E={\;\alpha }_{1}{\mathrm{RD}}_{\mathrm{it}}+{\;\alpha }_{2}{\mathrm{RECO}}_{\mathrm{it}}+{\;e}_{\mathrm{it}}$$

(2) $$\mathrm{CO}2E={\;\alpha }_{1}{\mathrm{RD}}_{\mathrm{it}}+{\;\alpha }_{2}{\mathrm{RECO}}_{\mathrm{it}}+\alpha_{3}{\mathrm{GDP}}_{\mathrm{it}}+{\;e}_{\mathrm{it}}$$

(3) $$\mathrm{CO}2E={\;\alpha }_{1}{\mathrm{RD}}_{\mathrm{it}}+{\;\alpha }_{2}{\mathrm{RECO}}_{\mathrm{it}}+\alpha_{3}{\mathrm{GDP}}_{\mathrm{it}}+\alpha_{4}{\mathrm{NRECO}}_{\mathrm{it}}+{\;e}_{\mathrm{it}}$$

(4) $$\mathrm{CO}2E={\;\alpha }_{1}{\mathrm{RD}}_{\mathrm{it}}+{\;\alpha }_{2}{\mathrm{RECO}}_{\mathrm{it}}+\alpha_{3}{\mathrm{GDP}}_{\mathrm{it}}+\alpha_{4}{\mathrm{NRECO}}_{\mathrm{it}}+\alpha_{5}{\mathrm{HCI}}_{\mathrm{it}}+{\;e}_{\mathrm{it}}$$

In the above equation, CO2E represents carbon emissions, R&D denotes research and development, GDP represents economic growth (taken as GDP per capita), RECO indicates renewable energy consumption (technologies-based). Likewise, i represents all G7C countries, t means the length of the time (1990–2020), all α’s denotes the parameters estimate, and e defines the stochastic term. Furthermore, all the variables in the above equation were converted to log form, including all control variables. However, scholars have examined the association between renewable energy consumption and CO2E without incorporating the main role of R&D in CO2E models. Our analysis would offer novel insights for policymakers by investigating the R&D-CO2E nexus incorporating the potential role of renewable energy and controlling economic growth and non-renewable energy consumption. The work of Wang, Zhang [33], and several other studies have revealed that improved renewable energy structure contributes to reducing CO2E, even in emerging economies.

## 4. Results and Discussion

### 4.1. Pre-Estimation Analysis

First, the results reported in Table 3 indicate that all models have problems with slope heterogeneity. Specifically, the probability value of the statistic in Table 2 is less than 5%, meaning that the model is facing the problem of slope heterogeneity. Next, we examine the presence of CSDP by applying Pesaran [55] method, which tests H0 (“No CSDP is present”) contrary to H1. To explain whether H0 is rejected or failed to reject, the results of Table 3 show that H0 of no CSDP is rejected for CO2E, R&D, RERC, GDP, and NRERC even at a 1% level.

Observing CSDP and slope heterogeneity helps us examine the level of stationarity of the series. We use Augmented Dickey-Fuller (CADF) and Cross-sectional lm, Pesaran, and Shin (CIPS) panel unit root tests to test the order of integration of all variables. Both are second-generation stationarity tests, though the second-generation panel unit tests offer similar outcomes. However, the findings of CIPS for the heterogeneous panel are more reliable than CADF and other first-generation panel unit root testing approaches. The CIPS test holds the single common factor that causes the assumption of cross-sectionally dependencies. The empirical value of both the tests (CADF and CIPS) is illustrated in Table 4. In the results (“except if there is an empty box”), the null hypothesis (H_0_) of the stationarity property is rejected for each series with level or first difference (mixed ordered). Specifically, the results suggest mixed order, though all stationarity tests offer the same outcomes. The results of the mixed-order panel unit root tests suggest applying the advanced panel cointegration test of Westerlund and CS-ARDL econometric model.

To tackle the issue of CSDP and other econometric problems, we employed the Westerlund [63] method. Table 5 shows the cointegration results between the variables, which is the evidence of different statistics (group statistics and panel group). Specifically, in Model 1, the long-run equilibrium between panel groups (P_t_ and P_a_) is statistically significant at a 1% level. For Model 2, the long-run equilibrium exists between overall groups (G_t_ and G_a_) and one panel (P_t_). The test statistic is statistically significant at the 1% level, and for Models 3 and 4, the test statistics are significant at 1% and 5%, respectively. This infers that long-run cointegration exists between groups and panels (G_t_, G_a_, P_t_, and P_a_).

### 4.2. Main Results

Table 6 provides the short and long-run results of CS-ARDL, where the response variable is CO2E. The empirical findings indicate that R&D, RNER, economic growth, and NRNER are all significant indicators to explain the variation in CO2E. The Error Correction Mechanism (ECM) supports the hypothetical association of the study between R&D and CO2E. The negative sign of the coefficient of R&D and renewable energy consumption implies that a rise in these factors helps to decrease CO2E in investigated countries. On the other hand, economic growth and nonrenewable energy consumption hold positive signs of coefficients, indicating a positive impact on the growth of CO2E. Specifically, the long-run elasticities (Equation (4), full model) of R&D, RENG consumption, economic growth, NRER are −0.091%, −0101%, 0.650%, and 0.138%, respectively.

The empirical results obtained from the CS-ARDL model provide many interesting points for discussion:

R&D negatively impacts CO2E in both the long and short run. Specifically, a 0.09% (Equation (4), full model) decrease in CO2E is caused by R&D-based projects in the long run. Furthermore, in the short run, a 0.08% reduction in CO2E is due to R&D. It infers that the clean environmental paradox in investigated economies could be attributed to the continuous R&D-based developmental process. The investigated economies enhance their environmental stability through developmental policy and innovative projects. The empirical results obtained here support the work of Ganda [64] and Petrović and Lobanov [41] for OECD countries.

On the other hand, the results are contrary to the work of [65,66]. According to Lee, Min [65], R&D can also contribute to increased CO2E, particularly in energy-intensive industries or fossil fuel use. This is because R&D often requires significant energy and resources, which can result in increased emissions if not managed effectively. The production and disposal of R&D equipment and materials can also contribute to emissions. However, the negative relationship between R&D and CO2E can be discussed in several ways;

R&D can help develop new technologies that reduce CO2E, improve energy efficiency, and promote renewable energy sources. At the same time, R&D can lead to new products and processes with lower CO2E [67]. Research and technologies-based renewable energy such as solar, wind, and hydropower can provide clean energy and reduce CO2E from traditional fossil fuel-based sources. R&D can lead to more efficient appliances, lighting, and building materials, and these improvements can reduce non-renewable energy consumption and CO2E [68]. In addition, R&D can lead to the development of low-emission or zero-emission vehicles that can reduce CO2E from transportation. At the same time, it can also help develop new technologies for carbon capture and storage [69].

By increasing the share of R&D (researchers per million), the inspected region could effectively begin many ecological protections and pollution mitigation projects. Raising the stakes of R&D and investment is the key to improving renewable energy and its raw material stock. It is the tool to increase the aggregate demand for renewable energy consumption. Furthermore, R&D investments are utilized to generate and explore environmental-friendly raw materials and fuels that can also make a critical and immediate positive impact on CO2E reduction. However, such progress in the R&D sector needs a good and cooperative relationship between policymakers, government authorities, industrialists, and academia.

It is noteworthy that, based on the CO2E base of each key industry in investigated countries, forecast emission reduction by 2060. The innovation and research direction process are the most significant support for carbon peaking and decline. It would promote the output and demonstration application of scientific and technological achievements and provide scientific and technical support for realizing the carbon reduction goal [41,70,71,72]. Overall, the Impact of R&D on CO2E depends on the specific technologies and products being developed. However, the trend revealed in recently published papers has been towards R&D that contributes to reducing carbon emissions and promoting sustainable development.

Another essential control variable (i.e., RENG), both the long-run and short-run elasticities (Equation (4), full model) of Table 6, suggest that RENG has a negative impact on the growth rate of CO2E. To be specific, a 0.101% long-run decline and 0.09% short-run decline in CO2E are caused by renewable energy consumption. The current study’s findings support the results of Khan, Yahong [10], Khan, Ali [60] for G7 countries, and Wang, Zhang [33] for 208 developing, emerging, and developed economies. The consumption of technology-based and advanced energy helps transform the conventional industrial sector into the green industrial sector. Moreover, the increasing per million researchers in developmental projects is vital in technological innovations and improving renewable energy supply structure. Thus, it can reduce overall CO2E and environmental instability [40,73].

The empirical outcomes further indicate that the coefficient of economic growth (i.e., GDP) positively impacts CO2E in both long and short-run estimates. The equation of Table 5 suggests that a 0.650% (long-run) and 0.700% (short-run) increase in CO2E is due to economic growth. A growth rate of economic activities exerts a significant burden on the intensity of CO2E and environmental instability. Thus, achieving a higher growth rate can reduce environmental stability in investigated countries. The current outcome on the relationship between economic growth and CO2E is similar to the work of Malik, Latif [61] and Khan and Yahong [74] for Pakistan, and Khan, Ali [60] and Khan, Yahong [10] for G7 economies.

Furthermore, the coefficient of nonrenewable energy (i.e., NRENG) negatively influences CO2E in the short and long run. On average, a 1% increase in NRENG will increase CO2E by approximately 0.138% (in the long run) and 0.136% (in the short run). Reducing the demand for nonrenewable energy consumption will help protect environmental quality and CO2E in the long and short run if a country lacks an efficient and advanced energy structure and cannot contribute to the carbon reduction goals set by international organizations. Therefore, improving the energy supply structure would significantly enhance environmental sustainability. The findings are consistent with the work of Murshed, Saboori [75] and Murshed, Saboori [75] for G-7 economies. Similarly, the work of Li, Wang [51] revealed the positive impact of non-renewable energy intensity on increasing the growth rate of CO2E in 147 developing, emerging, and developed economies, which is in line with the current results obtained here.

This study incorporated Dumitrescu and Hurlin [69] panel non-causality tests to test the causality between the variables. The empirical outcomes reported in Table 7 suggest uni-directional causality between R&D, economic growth, and NRENG with CO2E. In other words, the causal relationship between R&D, economic growth, human capital, and CO2E is not bi-directional, or there is no round effect among these variables. Furthermore, the round effect of bi-directional causality exists between RENG, human capital, and CO2E. However, the relationship between human capital and CO2E in Table 6 is insignificant across CS-ARDL models. For this reason, we can infer that the adaptation of any policy option for RENG and human capital has implications for CO2E.

For further analysis and robustness check, we incorporate the Augmented mean group (AMG) approach in this study. The empirical results obtained in Table 8 support the findings obtained in our primary model, Table 6. The findings indicate that the coefficient of all variables (except human capital) used across models (Equations (1)–(4)), such as rural development, renewable energy consumption, economic growth, and nonrenewable energy consumption, are important factors explaining variation in COE2. Although R&D-based developmental projects and RENG increases environmental sustainability regarding CO2E reduction.

## 5. Conclusions

The current study inspected how R&D, RENG, economic growth, and NRENG influenced CO2E in G-7 economies from 1990 to 2020. The Cs-Ardl (main model) and AMG (for robustness check) panel approaches were applied and examined the long-run and short-run impact of all independent variables (R&D, RENG, economic growth, and RENG) on the dependent variable (CO2E). According to the results, long-run R&D (research and development) and RENG (renewable energy) have a greater impact on reducing CO2E compared to the short run. Specifically, in the long run, the effect of R&D and RENG on reducing CO2E is −0.091 and −0.101, respectively, while in the short run, their impact is −0.084 and −0.094, respectively. On the other hand, economic growth measured by GDP per capita and NRENG (non-renewable energy) has harmful effects on CO2E. In particular, an increase of 0.650% (long run) and 0.700% (short-run) in CO2E is due to economic growth, while an increase of 0.138% (long run) and 0.136% (short-run) in CO2E is due to an increase in NRENG. These findings suggest that investing in long-term R&D and renewable energy can help reduce CO2E, while economic growth and non-renewable energy usage can negatively impact CO2E. It is important to note that these findings may vary depending on the specific context and the assumptions made in the analysis.

However, the result of human capital (HCI) was significantly negative but ineffective in CO2E reduction. Generally, it can be concluded that research-based activities, developmental projects (R&D), and green energy supply structures (RENG) in G7 economies currently affect CO2E inversely as they are more effective in protecting environmental sustainability. Therefore, it can be argued that the policymakers and governmental authorities of investigated countries must enhance environmental-based R&D to improve the energy supply structure. Doing so can increase green growth, reduce the demand for NRENG, and protect environmental quality.

Nevertheless, there is immense importance of the long-run and short-run policy options, enhancing the quality of environmental-based R&D and CO2E reduction that encourages green growth and sustainable development. Therefore, the concerned authorities should implement complete and suitable policies confirming that the investment in R&D and other developmental and technological processes must protect environmental stability. The governments of G-7 economies should also focus on human capital (as it has an insignificant role) to improve the economic property without the cost of environmental vulnerability. For this purpose, policymakers should educate general people and investors about the importance of environmental stability. In addition, it is crucial to make progressive reforms that only focus on green economic growth and ensure economic development without relying on RENG.

Furthermore, the current empirical work has great insights for concerned authorities, but not without policy limitations, which must be considered when conducting similar research. In this work, we utilized researcher per million and CO2E as indicators of technology-based R&D and environmental instability, respectively. It would be better if future studies used other measures of CO2E and R&D to verify the empirical results obtained here (whether it is robust or not). The current investigated the Impact of R&D and RENG on CO2E by incorporating the role of NRENG and economic growth in G7 economies. However, we provide the platform and empty gap for future researchers to examine similar issues in emerging or developing countries.

## Figures and Tables

**Table 1:** Related studies.

Studies on the R&D-CO2E Nexus				
Authors	Regions	Time	Methods	Results
Adedoyin, Alola [36]	28-European Union (EU) economies	1997–2014	DOLS and FMOLS	Negative
Alam, Apergis [37]	OECD	1996–2013	CCEMG	Negative
Churchill, Inekwe [38]	G7 economies	1870–2014	CCEMG	Negative
Fernández, López [39]	China, EU, and USA,	1990–2013	OLS	Negative
Mentel, Tarczyński [40]	26 developed and developing countries	1995–2015	FMOLS	Negative
Petrović and Lobanov [41]	OECD	1981–2014	OLS, AMG, and CCEMG	Negative
Yu and Xu [42]	China	2000–2017	Panel correlated Standard Error PCSE	Negative
Studies on the Renewable energy consumption-CO2E nexus				
Abbasi, Adedoyin [43]	Thailand	1980–2018	ARDL	Negative
Leitão and Lorente [44]	EU	1995–2014	DOLS, FMOLS, SGMM	Negative
Namahoro, Wu [45]	50 developing countries in Africa	1980–2018	CCEMG, PMG	Negative
Nathaniel and Iheonu [46]	19 developing countries in Africa	1990–2014	AMG	Negative
Obekpa and Alola [47]	United States	1974–2019	Breitung-candelon granger causality test	Mixed
Radmehr, Henneberry [48]	EU	1995–2014	GS2SLS	Negative
Wang, Zhang [33]	208 developed and developing countries	1990–2018	GMM and FMOLS	Negative
Studies on the economic growth-CO2E nexus				
Anwar, Younis [49]	East Asian region	1980–2017	VECM	Positive
Gorus and Aydin [50]	(MENA)	1975–2014	Granger causality	Positive
Li, Wang [51]	147 (mixed) economies	1990–2015	FMOLS and Granger causality	Positive
Studies on the nonrenewable energy-CO2E nexus				
Mujtaba, Jena [52]	OECD countries	1996–2016	ARDL	Positive
Fatima, Shahzad [53]	Eight high CO_2_ emitter economies	1980–2014	FE, GMM	Positive

Note. For the complete form of acronyms, please see the detailed table in Abbreviations.

**Table 2:** Details of the variables.

Symbols	Reference Paper	Unit of Measure	Hypothetical Sign
CO2E	Yahong, Cai [26], Wang, Wang [58]	Per-capita carbon emissions (matric tons)	
R&D	Kahouli [59]	Expenditures on Research and development expenditure (% of GDP)	Negative
RENG	Wang, Zhang [33]	Renewable energy consumption (% of total final energy consumption)	Negative
NRENG	Khan, Ali [60]	Industry (including construction), value added (% of GDP)	Positive
GDP	Malik, Latif [61], Wang, Yang [62]	GDP per capita (constant LCU)	Positive

**Table 3:** Slope heterogeneity and CSDP tests.

**Slope Heterogeneity**	
Statistics	**Value (*p*-Value)**
Delta	4.980 *** (0.000)
Adjusted delta	5.56 *** (0.000)
**CSDP tests**	
Variables	Statistic (*p*-value)
CO2E	19.556 *** (0.000)
R&D	20.099 *** (0.000)
RECO	23.842 *** (0.000)
NRERC	27.093 *** (0.000)
GDP	20.432 *** (0.000)

Note: Numerical values with three Asterisks (***) holds a 1% level of significance.

**Table 4:** CIPS and CADF unit root test.

Variables	Level		First Difference		Order
	C (Critical-Value)	C + T (Critical-Value)	C (Critical-Value)	C + T (Critical-Value)	
CIPS					
CO2E	−1.651 (−2.19)	−2.432 (−2.71)	−4.956 *** (−2.44)		Level
R&D	−2.222 ** (−2.19)	−3.164 ** (−2.86)			First
RENG	−1.884 (−2.19)	−3.245 ** (3.15)			First
GDP	−1.622 (−2.19)	−3.541 *** (−3.15)			First
NRENG	−1.835 (−2.19)	−2.557 (2.71)	−4.591 *** (−2.44)		Level
CADF					
CO2E	−1.857 (−2.210)	−2.248 (−2.270)	−5.079 *** (−2.570)		Level
R&D	−2.346 * (−2.210)	−2.821 * (−2.271)			First
RENG	−1.894 (−2.210	−3.063 *** (−3.060)			First
GDP	−1.621 (−2.210)	−2.875 ** (−2.840)			First
NRENG	−1.834 (−2.210)	−2.116 (−2.271)	−4.658 *** (−2.570)		Level

Note: Numerical values with three Asterisks (***), (**), and (*) hold a 1%, 5%, and 10% significance, respectively.

**Table 5:** Cointegration test.

Equations	G_t_	G_a_	P_t_	P_a_
Equation (1) [Z-value] (*p*-Value)	−3.069 *** [−2.349] (0.009)	−18.792 *** [−2.742] (0.003)	−5.157 [0.504] (0.693)	−8.105 [0.376] (0.647)
Equation (2) [Z-value] (*p*-Value)	−3.186 *** [−2.733] (0.003)	−17.876 *** [−2.378] (0.009)	−7.617 *** [−2.361] (0.009)	−9.532 [−0.256] (0.399)
Equation (3) [Z-value] (*p*-Value)	−4.062 *** [−5.620] (0.000)	−23.156 *** [−4.474] (0.000)	−7.080 ** [−1.735] (0.041)	−19.186 *** [−4.532] (0.000)
Equation (4) [Z-value] (*p*-Value)	−3.833 *** [−4.864] (0.000)	−16.252 * [−1.732] (0.041)	−6.791 * [−1.399] (0.081)	−15.694 * [−2.985] (0.001)

Note: Numerical values with three Asterisks (***), (**), and (*) hold a 1%, 5%, and 10% significance, respectively.

**Table 6:** CS-ARDL results.

Equation	1	2	3	4
	Coefficient. (S.d)	Coefficient. (S.d)	Coefficient. (S.d)	Coefficient. (S.d)
Variables	Long-run			
LnR&D	−0.136 ** (0.068)	−0.134 ** (0.079)	−0.195 ** (0.074)	−0.091 ** (0.068)
lnRENG	−0.173 ** (0.072)	−0.115 ** (0.064)	−0.111 ** (0.061)	−0.101 ** (0.058)
lnGDP		0.775 *** (0.189)	0.787 *** (0.188)	0.650 *** (0.216)
lnNRENG			0.153 *** (0.041)	0.138 *** (0.039)
lnHCI				0.626 (1.017)
ECM	−0.748 *** (0.073)	−0.7623 ** (0.059)	−0.765 *** (0.065)	−0.786 *** (0.061)
	Short-run			
LnR&D	−0.129 * (0.103)	−0.124 * (0.091)	−0.202 ** (0.096)	−0.084 ** (0.044)
lnRENG	−0.130 ** (0.081)	−0.129 ** (0.074)	−0.121 ** (0.059)	−0.094 ** (0.049)
lnGDP		0.699 *** (0.178)	0.689 *** (0.176)	0.700 *** (0.189)
lnNRENG			0.143 *** (0.036)	0.136 *** (0.040)
lnHCI				0.440 (1.199)

Note: Numerical values with three Asterisks (***), (**), and (*) hold a 1%, 5%, and 10% significance, respectively.

**Table 7:** Results of the non-causality test.

H_0_: There Is No Causality between Variables	Statistics		
	W-Bar	Z-Bar	*p*-Value
R&D ≠ CO2E	3.838 ***	3.663	0.000
CO2E ≠ R&D	2.110	1.465	0.120
RENG ≠ CO2E	5.919 ***	5.200	0.000
CO2E ≠ RENG	3.891 ***	3.515	0.009
GDP ≠ CO2E	4.999 ***	7.001	0.000
CO2E ≠ GDP	1.900	1.299	0.189
NRENG ≠ CO2E	5.956 ***	5.256	0.000
≠CO2E ≠ RENG	1.380	0.672	0.770
HCI ≠ CO2E	5.551 ***	5.201	0.000
CO2E ≠ HCI	2.961 ***	2.813	0.006

Note: Numerical values with three Asterisks (***) hold a 1% significance.

**Table 8:** AMG results.

Equations	1	2	3	4
	Coefficient. (S.d)	Coefficient. (S.d)	Coefficient. (S.d)	Coefficient. (S.d)
**Variables**	AMG			
lnRD	−0.176 ** (0.104)	−0.189 ** (0.090)	−0.180 ** (0.060)	−0.169 ** (0.058)
lnRECO	−0.174 ** (0.059)	−0.137 * (0.083)	−0.190 ** (0.089)	−0.158 * (0.095)
lnGDP		0.600 ** (0.265)	0.501 ** (0.220)	0.669 ** (0.249)
lnNRECO			0.185 * (0.101)	0.257 ** (0.105)
lnHCI				−0.305 (0.455)
Constant	−1.614 *** (0.512)	−1.039 *** (0.773)	−3.709 ** (1.993)	−5.783 *** (1.640)

Note: Numerical values with three Asterisks (***), (**), and (*) hold a 1%, 5%, and 10% significance, respectively.

## Data Availability

The data underlying the analysis in this study is available upon reasonable request (accessed on 1 March 2023).

## Abbreviations

Acronyms	
AMG	‘Augmented Mean Group’
ARDL	‘Autoregressive distributive lag’
CCEMG	‘Common correlated effects mean group’
DOLS	‘Dynamic ordinary least square’
EU	‘European Union
FE	‘Fixed Effect’
FMOLS	‘Fully modified OLS’
GMM	‘Generalized method of the moment
GS2SLS	‘Generalized spatial two-stage least squares
MENA	‘Middle East and North African’
OLS	‘Ordinary least square’
OECD	‘Organization for Economic Co-operation and Development
PCSE	‘Panel correlated Standard Error’
PMG	‘Pooled Mean Group’
SGMM	‘System-GMM’
VECM	‘Vector Error Correction Method’
